# The host response in critically ill sepsis patients on statin therapy: a prospective observational study

**DOI:** 10.1186/s13613-017-0349-3

**Published:** 2018-01-18

**Authors:** Maryse A. Wiewel, Brendon P. Scicluna, Lonneke A. van Vught, Arie J. Hoogendijk, Aeilko H. Zwinderman, René Lutter, Janneke Horn, Olaf L. Cremer, Marc J. Bonten, Marcus J. Schultz, Tom van der Poll

**Affiliations:** 1 Center for Experimental and Molecular Medicine, Academic Medical Center, University of Amsterdam, Meibergdreef 9, Room G2-130, 1105 AZ Amsterdam, The Netherlands; 2 The Center for Infection and Immunity Amsterdam, Academic Medical Center, University of Amsterdam, Meibergdreef 9, 1105 AZ Amsterdam, The Netherlands; 3 Department of Clinical Epidemiology, Bioinformatics, and Biostatistics, Academic Medical Center, University of Amsterdam, Meibergdreef 9, 1105 AZ Amsterdam, The Netherlands; 4 Department of Respiratory Medicine and Experimental Immunology, Academic Medical Center, University of Amsterdam, Meibergdreef 9, 1105 AZ Amsterdam, The Netherlands; 5 Department of Intensive Care, Academic Medical Center, University of Amsterdam, Meibergdreef 9, 1105 AZ Amsterdam, The Netherlands; 6 Department of Intensive Care Medicine, University Medical Center Utrecht, Heidelberglaan 100, 3584 CX Utrecht, The Netherlands; 7 Julius Center for Health Sciences and Primary Care, University Medical Center Utrecht, Heidelberglaan 100, 3584 CX Utrecht, The Netherlands; 8 Department of Medical Microbiology, University Medical Center Utrecht, Heidelberglaan 100, 3584 CX Utrecht, The Netherlands; 9 Division of Infectious Diseases, Academic Medical Center, University of Amsterdam, Meibergdreef 9, 1105 AZ Amsterdam, The Netherlands

**Keywords:** Statins, Sepsis, Host response, Biomarkers, Mortality

## Abstract

**Background:**

Statins can exert pleiotropic anti-inflammatory, vascular protective and anticoagulant effects, which in theory could improve the dysregulated host response during sepsis. We aimed to determine the association between prior statin use and host response characteristics in critically ill patients with sepsis.

**Methods:**

We performed a prospective observational study in 1060 patients admitted with sepsis to the mixed intensive care units (ICUs) of two hospitals in the Netherlands between January 2011 and July 2013. Of these, 351 patients (33%) were on statin therapy before admission. The host response was evaluated by measuring 23 biomarkers providing insight into key pathways implicated in sepsis pathogenesis and by analyzing whole-blood leukocyte transcriptomes in samples obtained within 24 h after ICU admission. To account for indication bias, a propensity score-matched cohort was created (*N* = 194 in both groups for protein biomarkers and *N* = 95 in both groups for gene expression analysis).

**Results:**

Prior statin use was not associated with an altered mortality up to 90 days after admission (38.0 vs. 39.7% in the non-statin users in the propensity-matched analysis). Statin use did not modify systemic inflammatory responses, activation of the vascular endothelium or the coagulation system. The blood leukocyte genomic response, characterized by over-expression of genes involved in inflammatory and innate immune signaling pathways as well as under-expression of genes associated to T cell function, was not different between patients with and without prior statin use.

**Conclusions:**

Statin therapy is not associated with a modified host response in sepsis patients on admission to the ICU.

**Electronic supplementary material:**

The online version of this article (10.1186/s13613-017-0349-3) contains supplementary material, which is available to authorized users.

## Background

Sepsis is the consequence of a deregulated host response to infection, featured by disproportionate pro- and anti-inflammatory mechanisms and disturbed vascular responses, including increased leukocyte adhesion, vasodilation, and loss of endothelial barrier function [1, 2]. In addition, obstruction of microvessel lumens by microthrombi and plugs of white and red blood cells, fibrin deposition and impaired anticoagulant mechanisms are other important elements of sepsis-induced organ dysfunction.

Statins, or HMG-CoA reductase inhibitors, are widely used to lower blood cholesterol levels. Besides decreasing cholesterol concentrations, statins have multiple additional effects that might influence the host response during sepsis, including inhibition of proinflammatory cytokine release and endothelial cell activation, reduction of endothelial dysfunction and attenuation of coagulation activation [3–6]. Several, but not all, observational studies have shown a survival benefit for patients with sepsis on statin therapy, with recent meta-analyses reporting an overall lower risk of sepsis and infection-associated death in chronic statin users [7, 8]. Considering the abundant literature on pleiotropic non-lipid lowering properties of statins, we investigated the association between prior statin use and potential host response alterations in this population of critically ill patients with sepsis. For this, we measured 23 biomarkers indicative of systemic inflammation, and activation of the vascular endothelium and the coagulation system, and in an unbiased approach analyzed whole-blood leukocyte transcriptomes in sepsis patients stratified according to prior statin use.

## Methods

### Study design, patients and definitions

This study was conducted as part of the “Molecular Diagnosis and Risk Stratification of Sepsis” (MARS) project, a prospective observational study in the mixed ICUs of two tertiary teaching hospitals (Academic Medical Center in Amsterdam and University Medical Center Utrecht) in the Netherlands [9–11]. Trained physicians prospectively collected the following data: demographics, comorbidities, chronic medication use, ICU admission characteristics, daily physiological measurements, severity scores, antibiotic use, and culture results. The plausibility of infection was post hoc scored based on all available evidence and classified on a 4-point scale (*none*, *possible*, *probable* or *definite*) according to Center for Disease Control and Prevention [15] and International Sepsis Forum consensus definitions [16], as described in detail previously [9]. For the current analysis, we selected all patients included in the MARS-study between January 2011 and July 2013 with sepsis, diagnosed within 24 h after admission, defined by the presence of a *definite* or *probable* infection [9] combined with at least one of general, inflammatory, hemodynamic, organ dysfunction or tissue perfusion parameters derived from the 2001 International Sepsis Definitions Conference [17]. Readmissions and patients transferred from another ICU were excluded, except for patients referred to one of the study centers on the day of admission. Organ failure was defined as a score of 3 or greater on the SOFA score, except for cardiovascular failure for which a score of 1 or more was used [12]. Shock was defined as use of vasopressors (noradrenaline) for hypotension in a dose of 0.1 mcg/kg/min during at least 50% of the ICU day. Patients were assessed daily for the presence of acute kidney injury and acute lung injury using strict preset criteria [13, 14]. Left-over plasma (obtained from blood drawn for patient care) was obtained within 24 h of admission to the ICU and stored within 4 h at − 80 °C. The Medical Ethical Committees of both study centers gave approval for an opt-out consent method (IRB no. 10-056C) [9, 10]. The Municipal Personal Records Database was queried to determine survival up to 1 year after ICU admission.

### Biomarker assays

All measurements were performed in EDTA anticoagulated plasma obtained on admission. Tumor necrosis factor alpha (TNF-α), interleukin-1beta (IL-1β), IL-6, IL-8, IL-10, IL-13, interferon-γ, granulocyte-macrophage colony-stimulating factor (GM-CSF), soluble intercellular adhesion molecule-1 (ICAM-1), soluble E-selectin and fractalkine were measured using FlexSet cytometric bead arrays (BD Bioscience, San Jose, CA) using a FACS Calibur (Becton Dickenson, Franklin Lakes, NJ, USA). Angiopoietin-1, angiopoietin-2, protein C, antithrombin, matrix metalloproteinase (MMP)-8, tissue inhibitor of metalloproteinase (TIMP)-1 (R&D systems, Abingdon, UK), and D-dimer (Procartaplex, eBioscience, San Diego, CA) were measured by Luminex multiplex assay using a BioPlex 200 (BioRad, Hercules, CA). C-reactive protein (CRP) was determined by an immunoturbidimetric assay (Roche diagnostics). Platelet counts were determined by hemocytometry, prothrombin time (PT) and activated partial thromboplastin time (aPTT) by using a photometric method with Dade Innovin Reagent or by Dade Actin FS Activated PTT Reagent, respectively (both Siemens Healthcare Diagnostics). Normal biomarker values were acquired from 27 age- and gender-matched healthy volunteers, from whom written informed consent was obtained, except for CRP, platelet counts, PT and aPTT (routine laboratory reference values).

### Blood gene expression microarrays

Whole blood was collected in PAXgene™ tubes (Becton–Dickinson, Breda, the Netherlands) within 24 h after ICU admission. PAXgene blood samples were also obtained from 42 healthy controls [median age 35 (interquartile range 30–63) years; 57% male] after providing written informed consent. Total RNA was isolated using the PAXgene blood mRNA kit (Qiagen, Venlo, the Netherlands) in combination with QIAcube automated system (Qiagen, Venlo, the Netherlands), according to the manufacturer’s instructions. RNA (RNA integrity number > 6.0) was processed and hybridized to the Affymetrix Human Genome U219 96-array and scanned by using the GeneTitan instrument at the Cologne Center for Genomics (CCG), Cologne, Germany, as described by the manufacturer (Affymetrix).

Raw data scans (.CEL files) were read into the R language and environment for statistical computing (version 2.15.1; R Foundation for Statistical Computing, Vienna, Austria; http://www.R-project.org/). Pre-processing and quality control was performed by using the Affy package version 1.36.1. Array data were background corrected by robust multi-array average, quantiles-normalized and summarized by median polish using the expresso function (Affy package). The resultant 49,386 log-transformed probe intensities were filtered by means of a 0.5 variance cutoff using the genefilter method [18] to recover 24,646 expressed probes in at least one sample. The occurrence of non-experimental chip effects was evaluated by means of the Surrogate Variable Analysis (R package version 3.4.0) and corrected by the empirical Bayes method ComBat [19, 20]. The non-normalized and normalized MARS gene expression data sets are available at the Gene Expression Omnibus public repository of NCBI under accession number GSE65682. The 24,646 probes were assessed for differential abundance across healthy subject and patient samples by means of the limma method (version 3.14.4) [21]. Supervised analysis (comparison between pre-defined groups) was performed by moderated t statistics. Throughout Benjamini–Hochberg (BH) multiple comparison adjusted probabilities, correcting for the 24,646 probes (false discovery rate < 5%), defined significance. Ingenuity Pathway Analysis (Ingenuity Systems IPA, http://www.ingenuity.com) was used to identify the associating canonical signaling pathways stratifying genes by over- and under-expressed patterns. The ingenuity gene knowledgebase was selected as reference and human species specified. All other parameters were default. Multiple comparison adjusted Fisher test probabilities < 0.05 defined significance.

### Statistical analysis

Data analyses were performed in R (v3.1.1) [22]. Baseline characteristics of study groups were compared with Chi-square test for categorical variables and t-test for continuous variables. Non-normally distributed continuous variables, including biomarker levels, were analyzed with Wilcoxon rank sum test. To account for differential likelihood of receiving statins, we constructed a propensity score [23], using logistic regression, including variables associated with use of statins and variables that we considered of relevance to our outcome. This score included age, gender, weight, race (white), cerebrovascular disease, chronic cardiovascular insufficiency, chronic renal insufficiency, congestive heart failure, chronic obstructive pulmonary disease (COPD), diabetes mellitus, hematologic malignancy, hypertension, metastatic malignancy, history of myocardial infarction, ACE-inhibitors/ARBs, antiplatelet drugs, beta-blockers, oral antidiabetic drugs, and site of infection (pulmonary, abdominal, urinary). Subjects were 1:1 matched by the estimated propensity score using nearest neighbor matching with a caliper of 0.2SD of the logit of the propensity score, using R package “MatchIt”. Patients whose plasma samples were not collected for biomarker analyses within 24 h of ICU admission and were excluded from the matching procedure. In addition, matching for analyses of gene expression profiles was done using only patients from whom gene expression data were available. Standardized differences were calculated to determine balance between the propensity-matched groups [24]. In order to retain enough power to detect differences in biomarker levels, we accepted standardized differences between propensity-matched groups for comorbidities and chronic medication up to 20%. To investigate the independent association between statin use and 30-day mortality in our propensity-matched plasma biomarker cohort, we performed logistic regression including statin use, variables associated with mortality and comorbidities not optimally matched between users and non-users. *P* values below 0.05 were considered statistically significant. In host response biomarker comparisons, a Bonferroni-corrected *P* value of 0.002 was taken as cutoff to define statistical significance.

## Results

### Study population

From January 2011 until July 2013, 6994 admissions were included in the MARS-study, of which 1483 involved an admission diagnosis of sepsis (Additional file 1: Figure 1). Transfers from other ICUs and readmissions were excluded (129 and 250, respectively). Prior use of medication could not be traced in 44 cases. As a result, 1060 patients were included for analysis, of whom 351 (33.1%) used statins (Table 1). Simvastatin was the most common statin prescribed (53.8%), followed by atorvastatin (21.4%), pravastatin (14%) and rosuvastatin (8%). Patients who used statins were older, more frequently men, and had higher body mass indexes. As expected, statin users were more often suffering from diabetes, hypertension, cerebrovascular disease, chronic renal insufficiency, congestive heart failure, COPD and peripheral vascular disease; statin users had a lower prevalence of malignancy. In accordance with these differences in comorbid conditions, statin users more often used a variety of other types of chronic medication, including ACE inhibitors, ARBs, antiplatelet drugs, beta-blockers, insulin, and oral antidiabetic drugs. Statin use was associated with a lower prevalence of alcohol or drug abuse. Considering the large differences in demographics and comorbidities between users and non-users of statins at baseline, we constructed propensity score-matched cohorts to correct for these pre-admission dissimilarities [23]. Nine patients (1%) could not be assigned a propensity score due to missing data. In total, 194 of 351 statin users could be matched to non-users (Table 1 and Additional file 1: Figure 2). Yet, a higher prevalence peripheral vascular disease and use of antiplatelet drugs remained in the statin group after propensity score matching.

**Table 1:** Baseline characteristics of sepsis patients admitted to the ICU stratified according to prior use of statins

Characteristics	Unmatched cohort			Propensity-matched cohort		
	Statins *N* = 351	No statins *N* = 709	*p*	Statins *N* = 194	No statins *N* = 194	*p*
*Demographics*						
Age, years, mean [SD]	67.0 [9.9]	58.7 [15.6]	< .0001	66.7 [10.5]	65.8 [13.2]	.43
Gender, male (%)	238 (67.8)	402 (56.7)	.002	123 (63.4)	121 (62.4)	.93
Race, white (%)	315 (89.7)	619 (87.3)	.19	175 (90.2)	175 (90.2)	1
BMI, kg/m^2^, mean [SD]	26.8 [6.2]	25.6 [6.1]	.002	26.6 [6.09]	27.0 [6.95]	.49
*Comorbidities*						
Cerebrovascular disease (%)	58 (16.5)	43 (6.1)	< .001	32 (16.5)	24 (12.4)	.31
Chronic cardiovascular insufficiency (%)	22 (6.3)	17 (2.4)	.002	11 (5.7)	10 (5.2)	1
Chronic renal insufficiency (%)	88 (25.1)	67 (9.4)	< .001	46 (23.7)	38 (19.6)	.38
Congestive heart failure (%)	29 (8.3)	23 (3.2)	.002	10 (5.2)	10 (5.2)	1
COPD (%)	70 (19.9)	89 (12.6)	.005	32 (16.5)	36 (18.6)	.70
Diabetes mellitus (%)	133 (37.9)	89 (12.6)	< .001	60 (30.9)	48 (24.7)	.19
Hematologic malignancy (%)	9 (2.6)	70 (9.9)	.001	7 (3.6)	6 (3.1)	1
Hypertension (%)	174 (49.6)	159 (22.4)	< .001	81 (41.8)	76 (39.2)	.68
Immune deficiency (%)	72 (20.5)	158 (22.3)	.52	38 (19.6)	43 (22.2)	.63
Metastatic malignancy (%)	6 (1.7)	39 (5.5)	.004	3 (1.5)	1 (0.5)	.62
Myocardial infarction (history of) (%)	70 (19.9)	30 (4.2)	< .001	29 (14.9)	22 (11.3)	.38
Non-metastatic malignancy (%)	62 (17.7)	89 (12.6)	.03	43 (22.2)	33 (17)	.24
Peripheral vascular disease (%)	82 (23.4)	50 (7.1)	< .001	46 (23.7)	26 (13.4)	.01
Alcohol or drug abuse (%)	17 (4.8)	60 (8.5)	.04	11 (5.7)	17 (8.8)	.33
*Chronic medication*						
ACE inhibitors and ARBs (%)	192 (54.7)	134 (18.9)	< .001	90 (46.4)	76 (39.2)	.19
Anticoagulants (%)	72 (20.5)	97 (13.7)	.009	45 (23.2)	44 (22.7)	1
Antiplatelet drugs (%)	203 (57.8)	91 (12.8)	< .001	91 (46.9)	69 (35.6)	.03
Beta-blockers (%)	215 (61.3)	135 (19)	< .001	100 (51.5)	86 (44.3)	.18
Calcium channel blockers (%)	108 (30.8)	79 (11.1)	< .001	54 (27.8)	46 (23.7)	.42
Corticosteroids (%)	54 (15.4)	109 (15.4)	1	28 (14.4)	37 (19.1)	.27
Insulin (%)	77 (21.9)	51 (7.2)	< .001	43 (22.2)	28 (14.4)	.05
Oral antidiabetic drugs (%)	91 (25.9)	47 (6.6)	< .001	38 (19.6)	27 (13.9)	.18
Other antiarrhythmic drugs (%)	27 (7.7)	28 (3.9)	.008	17 (8.8)	17 (8.8)	1
Statins						
Simvastatin (%)	189 (53.8)	–		109 (56.2)	–	
Atorvastatin (%)	75 (21.4)	–		37 (19.1)	–	
Pravastatin (%)	49 (14)	–		27 (13.9)	–	
Rosuvastatin (%)	28 (8)	–		15 (7.7)	–	
Fluvastatin (%)	8 (2.3)	–		4 (2.1)	–	
Unknown statin (%)	2 (0.6)	–		2 (1)	–	
*Site of infection*						
Pulmonary (%)	137 (39)	326 (46)	.04	75 (38.7)	79 (40.7)	.77
Abdominal (%)	63 (17.9)	140 (19.7)	.50	39 (20.1)	31 (16)	.37
Urinary tract (%)	45 (12.8)	64 (9)	.07	25 (12.9)	24 (12.4)	1
Other (%)^a^	64 (18.2)	101 (14.2)	.11	33 (17)	38 (19.6)	.60
Co-infection (%)	42 (12)	78 (11)	.69	22 (11.3)	22 (11.3)	1
Admission type, medical (%)	253 (72.1)	531 (74.9)	.34	135 (69.6)	155 (79.9)	.02
*Causative pathogens* ^b^						
Gram-positive (%)	184 (52.4)	327 (46)	.34	88 (45.4)	85 (43.8)	.79
Gram-negative (%)	220 (62.7)	395 (55.7)	.31	119 (61.3)	111 (57.2)	.49
Yeast/fungi (%)	38 (10.8)	79 (11.1)	.68	20 (10.3)	25 (12.9)	.54
Other (%)	39 (11.1)	94 (13.3)	.21	25 (12.9)	24 (12.4)	.89
Unknown (%)	51 (14.5)	124 (17.5)	.14	26 (13.4)	34 (17.5)	.35
*Severity of disease in first 24* *h*						
APACHE IV Score, median [IQR]	83 [67–103]	78 [61–101]	.04	85 [66–103]	83 [66–106]	.95
Acute physiology score, median [IQR]	68 [51–86]	65 [51–85]	.45	71 [52–87]	67 [53–92]	,91
SOFA score, median [IQR]^c^	8 [6–10]	7 [5–9]	.007	8 [6–10]	7 [5–9.75]	.38
Organ failure (%)	295 (84)	600 (84.6)	.09	169 (87.1)	174 (89.7)	.79
Shock (%)	119 (33.9)	240 (33.9)	1	73 (37.6)	77 (39.7)	.75
Acute lung injury (%)	89 (25.4)	202 (28.5)	.30	53 (27.3)	50 (25.8)	.82
Acute kidney injury (%)	157 (44.7)	271 (38.2)	.04	81 (41.8)	90 (46.4)	.41
Mechanical ventilation (%)	272 (77.5)	549 (77.4)	1	153 (78.9)	155 (79.9)	.90
Renal replacement therapy (%)	48 (13.7)	61 (8.6)	.02	32 (16.5)	19 (9.8)	.05
Lactate max. (mmol/l), median [IQR]^d^	2.6 [1.7–4.9]	2.6 [1.6–4.77]	.57	2.5 [1.6–4.6]	2.9 [1.8–4.6]	.41

*ACE* angiotensin-converting-enzyme, *APACHE* acute physiology and chronic health evaluation, *ARBs* angiotensin receptor blockers, *BMI* body mass index, *COPD* chronic obstructive pulmonary disease, *IQR* interquartile range, *NSAIDs* non-steroidal anti-inflammatory drugs, *SD* standard deviation, *SOFA* sequential organ failure assessment

^a^Site of infection: “other” includes cardiovascular infection, mediastinitis and skin infection

^b^Percentages represent the proportion of cases caused by the particular pathogen. In some cases multiple causative pathogens were isolated

^c^Central nervous system not included in score, due to large number of sedated patients

^d^Lactate levels were absent in 220 patients

### Statin use and sepsis presentation and outcome

In the unmatched comparison, statins users presented with higher median APACHE IV (median 83 vs. 78, *P* = 0.04) and SOFA scores (median 8 vs. 7, *P* = 0.007). Acute kidney injury was more frequently observed in statin users (44.7%) compared to non-users (38.2%, *P* = 0.04) and renal replacement therapy more often required (13.7 vs. 8.6%, *P* = 0.02). Sites of infection were largely similar between groups, besides a pulmonary source of infection, which was less frequently recorded in the statin group (39.0 vs. 46.0%, *P* = 0.04). Following propensity score matching on pre-admission variables, none of these differences in sepsis presentation and severity were present anymore.

Statin users were similar to non-users with regard to ICU or hospital length of stay, development of ICU-acquired complications or mortality up to up to 90 days after ICU admission, in either the unmatched or the matched cohort (Table 2). The association of statin use with 30-day mortality was further studied using logistic regression in the propensity-matched cohort, which revealed a survival benefit for prior statin users (odds ratio 0.58, 95% confidence intervals 0.36–0.93; Table 3).

**Table 2:** Outcomes of sepsis patients admitted to the ICU stratified according to prior use of statins

Outcomes	Unmatched cohort			Propensity-matched cohort		
	Statins *N* = 351	No statins *N* = 709	*p*	Statins *N* = 194	No statins *N* = 194	*p*
Length of stay ICU, median, days [IQR]	4 [2–9]	5 [2–10]	.28	4 [2–11]	5 [2–11]	.58
Organ failure during admission (%)	307 (87.5)	634 (89.4)	.32	175 (90.2)	180 (92.8)	1
Shock during admission (%)	150 (42.7)	296 (41.7)	.80	93 (47.9)	97 (50)	.76
Acute lung injury during admission (%)	107 (30.5)	232 (32.7)	.49	66 (34)	55 (28.4)	.29
Acute kidney injury during admission (%)	183 (52.1)	327 (46.1)	.07	99 (51)	106 (54.6)	.53
Mortality						
ICU mortality (%)	65 (18.5)	149 (21)	.36	35 (18)	48 (24.7)	.13
Hospital mortality (%)	106 (30.2)	226 (31.9)	.58	62 (32)	73 (37.6)	.31
30-day mortality (%)	92 (26.2)	198 (27.9)	.60	48 (24.7)	67 (34.5)	.051
60-day mortality (%)	112 (31.9)	235 (33.1)	.71	61 (31.4)	74 (38.1)	.24
90-day mortality (%)	129 (36.8)	255 (36)	.84	74 (38.1)	77 (39.7)	.83

*ICU* intensive care unit, *IQR* interquartile range

**Table 3:** Association of statin use with 30-day mortality using logistic regression in propensity-matched cohort

	Odds ratio	95% confidence interval	*p*
Statins	0.58	0.36–0.93	.02
APACHE IV score	1.02	1.01–1.03	< .0001
Age	1.04	1.01–1.06	.002
Hematologic malignancy	1.61	0.46–5.64	.45
Non-metastatic malignancy	0.93	0.52–1.66	.79
Peripheral vascular disease	1.93	1.09–3.44	.02
Diabetes mellitus	0.89	0.53–1.51	.68

### Statin use and systemic host response biomarkers

**Fig. 1:**
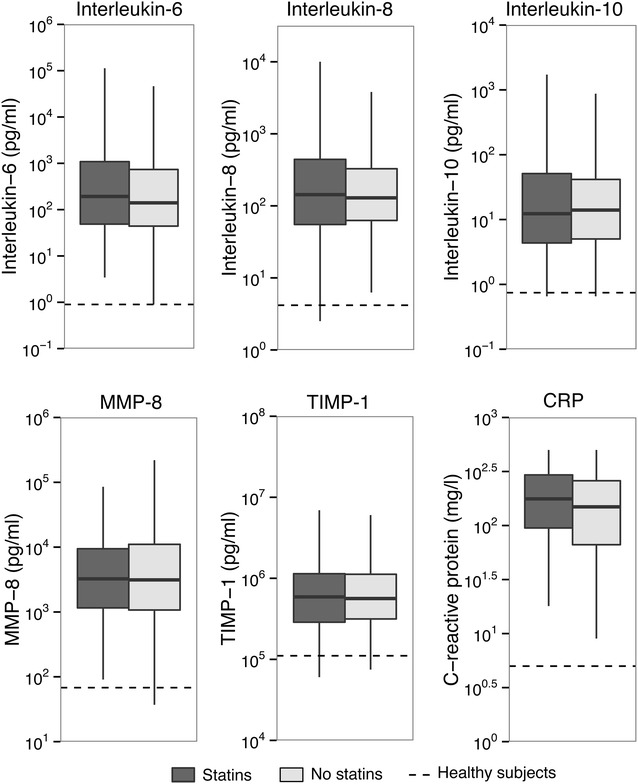
Inflammatory responses in sepsis patients on ICU admission stratified according to statin use in the propensity-matched cohort. Data are expressed as box-and-whisker diagrams depicting the median and lower quartile, upper quartile and their respective 1.5IQR as whiskers (as specified by Tukey). CRP levels were missing in 104 cases. Differences between groups were not significant. Dashed lines represent median levels in 27 healthy volunteers

**Fig. 2:**
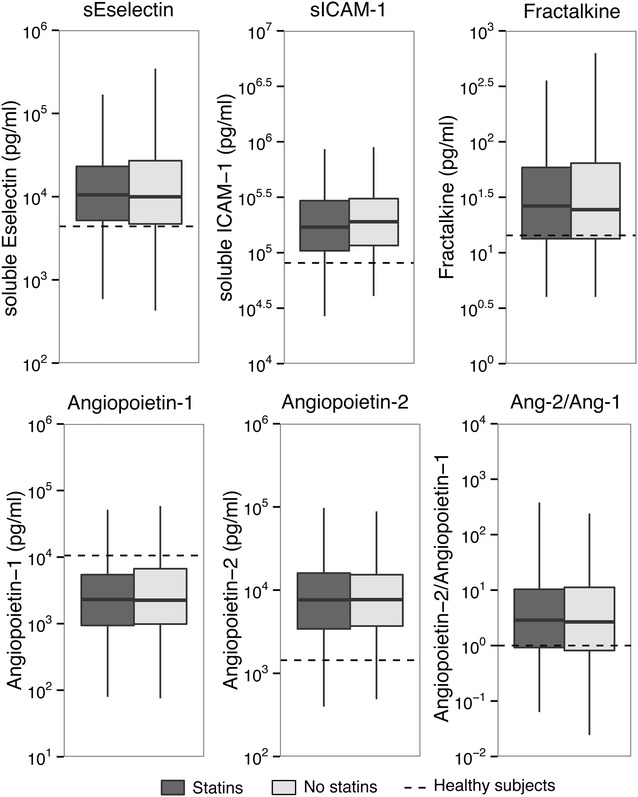
Endothelial cell activation in sepsis patients on ICU admission stratified according to statin use in the propensity-matched cohort. Data are expressed as box-and-whisker diagrams depicting the median and lower quartile, upper quartile and their respective 1.5IQR as whiskers (as specified by Tukey). Dashed lines represent median levels in 27 healthy volunteers. Differences between groups were not significant. ICAM-1 = intercellular adhesion molecule-1

**Fig. 3:**
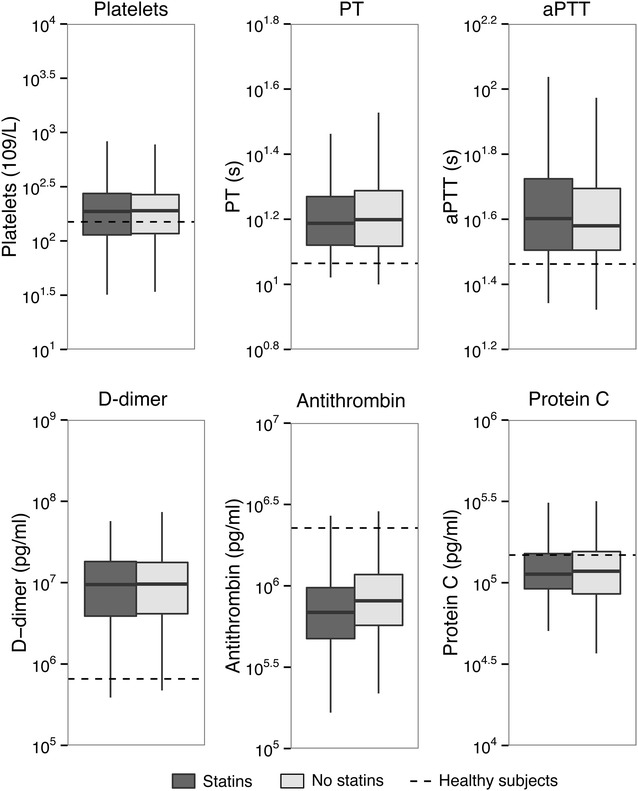
Coagulation activation in sepsis patients on ICU admission stratified according to statin use in the propensity-matched cohort. Data are expressed as box-and-whisker diagrams depicting the median and lower quartile, upper quartile and their respective 1.5IQR as whiskers (as specified by Tukey). Dashed lines represent median levels in 27 healthy volunteers, except for platelets, prothrombin time and activated partial thromboplastin time, which represents the clinical laboratory lower and upper reference values, respectively. APTT was missing in 127 cases, PT in 10 and platelet count in 1 patient. Differences between groups were not significant

We measured 23 biomarkers indicative of host response pathways implicated in sepsis pathogenesis in plasma or blood obtained < 24 h after ICU admission (Additional file 1: Table 1 for unmatched cohort; Figures 1–3 for matched cohort). Relative to healthy controls, patients with sepsis displayed signs of systemic inflammation, as reflected by a profound activation of the cytokine network (elevated plasma levels of IL-6, IL-8 and IL-10), elevated levels of MMP-8 and TIMP-1 and an increased acute phase protein response (elevated plasma CRP concentrations) (Fig. 1). In addition, sepsis was associated with activation of the vascular endothelium (elevated plasma concentrations of soluble E-selectin, soluble ICAM-1, fractalkine and angiopoietin-2, and reduced levels of angiopoietin-1) (Fig. 2) and the coagulation system (elevated D-dimer levels, prolonged PT and aPTT, and reduced levels of the anticoagulant proteins protein C and antithrombin) (Fig. 3). Platelet counts were not significantly altered in patients with sepsis relative to healthy controls.

None of these responses differed between statin users and statin non-users, in either the unmatched cohort (Additional file 1: Table 1) or the matched cohort (Figs. 1, 2, 3). The plasma concentrations of TNF-α, interferon-γ, IL-1β, IL-13 and GM-CSF were undetectable or very low in the vast majority of patients and not different between groups (data not shown).

### Statin use and the blood leukocyte genomic response

**Fig. 4:**
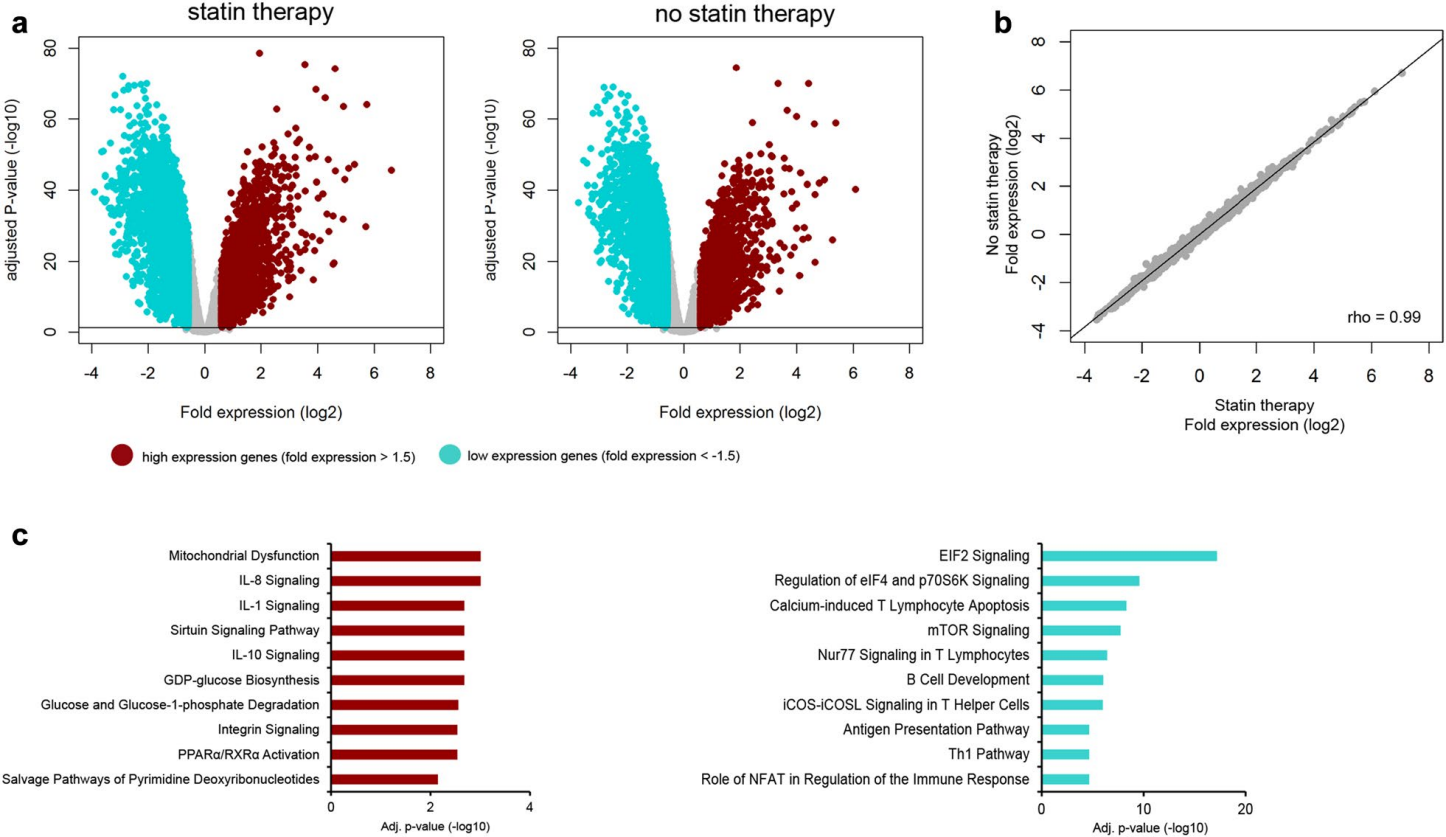
Blood transcriptomics of sepsis patients discordant for statin therapy in the propensity-matched cohort. **a** Volcano plot representation of differential gene expression in patients treated with statins (*N* = 95) and patients not treated with statins (*N* = 95), both relative to healthy subjects. **b** Dot plot illustrating the strong correlation between expression changes in sepsis patients discordant for statin therapy, relative to healthy subjects. Rho, Spearman’s rho. **c** Ingenuity pathway analysis of genes with elevated expression (red bars) and decreased expression (turquoise) (*N* = 190)

Using an unbiased approach we compared the blood leukocyte transcriptome of sepsis patients who were on statin therapy (*N* = 157) versus those who were not (*N* = 337). With this method, we studied gene expression genome-wide (i.e., contrasting with a biased approach in which a particular signaling pathway is studied). This analysis comprised the subgroup of patients enrolled during the first 1.5 years of this study. At first, genome-wide blood gene expression profiles of statin users and statin non-users were compared to 42 healthy controls. Pronounced alterations in gene expression were detected in both patient groups, which were strongly correlated (Additional file 1: Figure 3). Elevated expression of genes involved in typical pro-, anti-inflammatory, innate immune and metabolic pathways concomitant with decreased expression of predominantly T cell signaling pathways characterized this previously reported common host response [11]. Comparing the leukocyte transcriptomes of patients with statin therapy to those patients who did not revealed no statistically significant differences. We subsequently compared leukocyte transcriptomes of patients in the matched cohort [statin therapy (*N* = 95) and no statin therapy (*N* = 95)] (Fig. 4). Clinical characteristics of matched patients are shown in Additional file 1: Tables 2 and 3. Again, similar alterations in leukocyte transcriptomes of both patient groups were uncovered relative to health, with strongly correlated gene expression changes. No differences in leukocyte transcriptomes were uncovered when comparing patients with statin therapy to those patients who did not receive statin therapy in this matched cohort.

## Discussion

In a number of randomized trials in infectious/inflammatory conditions such as ventilator-associated pneumonia and acute respiratory distress syndrome, conducted over the past years, statins failed to improve outcome [25–27]. The majority of earlier observational studies, however, reported improved outcome of statin users with sepsis [7, 8]. In accordance, by using logistic regression analysis in the propensity-matched cohort, we found a survival benefit for prior statin users. The primary objective of this study was to compare the host response between prior users and non-users of statins in sepsis patients upon admission to the ICU. We measured 23 biomarkers, providing insight into systemic inflammatory reactions, activation of the endothelium and the coagulation system, and studied whole genome expression profiles in blood leukocytes, and compared these between sepsis patients who were on statin therapy prior to admission and those who were not, in both an unmatched and a propensity score-matched cohort. We defined sepsis using the 2001 consensus definition [17]; the vast majority of MARS patients included in this analysis had a SOFA score ≥ 2 at ICU admission, which approximates the most recent consensus definitions for sepsis [28]. Our results strongly suggest that prior statin therapy does not influence the host response to sepsis in patients requiring intensive care.

Previous studies in patients with infection and/or sepsis reporting on an association between statin use and host response biomarkers were small or limited to a few biomarkers. To our knowledge, only one earlier study focused on sepsis patients admitted to the ICU: in a randomized trial of 250 critically ill patients with severe sepsis, prior statin users had lower baseline levels of IL-6 compared to statin-naïve patients; treatment with atorvastatin during admission did not alter IL-6 levels compared to placebo in either prior statin users or statin-naïve patients [29]. In a targeted approach, we measured a series of biomarkers that were selected because they provide insight into host response pathways implicated in the pathogenesis of sepsis [1, 2] and because statins have been shown to exert inhibitory effects on these mechanisms [3–6]. None of the biomarkers determined were different between prior statin users and non-users. Our results are in accordance with a study in 1895 patients with community-acquired pneumonia, in whom prior statin use did not influence cytokine release or coagulation activation, except for a modest increase in antithrombin levels [30]. This latter study is different from our cohort, as it was conducted in emergency departments with less than 20% of patients requiring intensive care and only encompassed patients with community-acquired pneumonia. Two smaller investigations in non-ICU patients reported on the association between statin use and the host response during suspected or documented infection: in a randomized trial involving 84 hospitalized patients who were not using statins prior to admission TNF-α and IL-6 levels were significantly reduced in patients after treatment with simvastatin [31]; in an observational study in 209 hospitalized patients prior statin use was not associated with altered C-reactive protein levels upon admission [32]. Taken together, these and our study suggest that statin use prior to admission has little if any impact on the host response to infection in patients admitted to either a general hospital ward or the ICU.

Statins have been reported to modulate the host response in controlled models of human inflammation induced by intravenous or intrabronchial administration of lipopolysaccharide (LPS). Simvastatin attenuated proinflammatory cytokine release, procoagulant responses and vascular hyporeactivity induced by intravenous LPS injection into healthy humans [33, 34], and reduced neutrophil influx and the release of myeloperoxidase, TNF-α and metalloproteinases (including MMP-9) in bronchoalveolar lavage fluid after an intrabronchial challenge with LPS [35]. While these data are in accordance with the immune modulatory properties of statins in various experimental settings [3–6], our results indicate that the potential anti-inflammatory and anticoagulant effects of statins do not influence the rigorous and unbalanced host response in a heterogeneous population of critically ill patients with sepsis.

This study has limitations. First, our study was observational; the findings cannot prove cause and effect. Second, this study was underpowered to detect small differences; nevertheless, the clinical relevance of such minor differences would be unclear. Third, although propensity score matching is an elegant way to adjust for multiple baseline differences between the investigational groups, bias can occur as a result of unmeasured confounders. Furthermore, unbalanced clinical baseline conditions remained in our propensity-matched cohort; however, in separate analyses diabetes, oral antidiabetic or antiplatelet drugs did not influence sepsis outcome or host response [36, 37]. Samples from healthy volunteers were taken as controls for biomarker analysis; hence, the change in biomarker levels cannot be specifically attributed to sepsis but may, in part, be related to an inflammatory response to acute severe disease. Although we have determined a variety of systemic host response protein biomarkers, aiming to characterize relevant pathways in sepsis pathogenesis, some biomarkers of interest were not measured, including those providing insight in the function of the glycocalyx. An additional limitation is the lack of information about the duration and adherence to statins. Strengths of our study are its prospective nature, in which consecutively admitted patients were included and disease presentation, course and outcome were meticulously documented.

In conclusion, prior statin therapy was not associated with an altered host response in patients with sepsis upon admission to the ICU.

## Additional file

**Additional file 1: Figure 1.** Flowchart of patients. **Figure 2.** Distribution of propensity scores. **Figure 3.** Blood transcriptomics of sepsis patients discordant for statin therapy. **Table 1.** Host response biomarkers in sepsis patients admitted to the ICU stratified according to prior use of statins in the unmatched cohort. **Table 2.** Baseline characteristics of matched statin-users and non-users in the gene expression study. **Table 3.** Outcomes of matched statin-users and non-users in the gene expression study.

## Abbreviations

APACHE: acute physiology and chronic health evaluation
aPTT: activated partial thromboplastin time
COPD: chronic obstructive pulmonary disease
CRP: C-reactive protein
ICAM: intercellular adhesion molecule
ICU: intensive care unit
IL: interleukin
GM-CSF: granulocyte-macrophage colony-stimulating factor
MMP: matrix metalloproteinase
PT: prothrombin time
SOFA: sequential organ failure assessment
TIMP: tissue inhibitor of metalloproteinase
TNF: tumor necrosis factor
