# Supplementary material for: The host response in critically ill sepsis patients on statin therapy: a prospective observational study
Source: Ann Intensive Care. 2018 Jan 18;8:9. doi: 10.1186/s13613-017-0349-3 (PMC5773465; doi:10.1186/s13613-017-0349-3)
Supplement: Supplementary file 1 — Additional file 1: Figure 1. Flowchart of patients. Figure 2. Distribution of propensity scores. Figure 3. Blood transcriptomics of sepsis patients discordant for statin therapy. Table 1. Host response biomarkers in sepsis patients admitted to the ICU stratified according to prior use of statins in the unmatched cohort. Table 2. Baseline characteristics of matched statin-users and non-users in the gene expression study. Table 3. Outcomes of matched statin-users and non-users in the gene expression study. [file 13613_2017_349_MOESM1_ESM.docx]

**Additional file**

**The host response in critically ill sepsis patients on statin therapy;**

**a prospective observational study**

Maryse A Wiewel, Brendon P Scicluna, Lonneke A van Vught, Arie J Hoogendijk,

Aeilko H Zwinderman, René Lutter, Janneke Horn, Olaf L Cremer, Marc J Bonten,

Marcus J Schultz, and Tom van der Poll

**Supplemental Figure 1. Flowchart of patients**


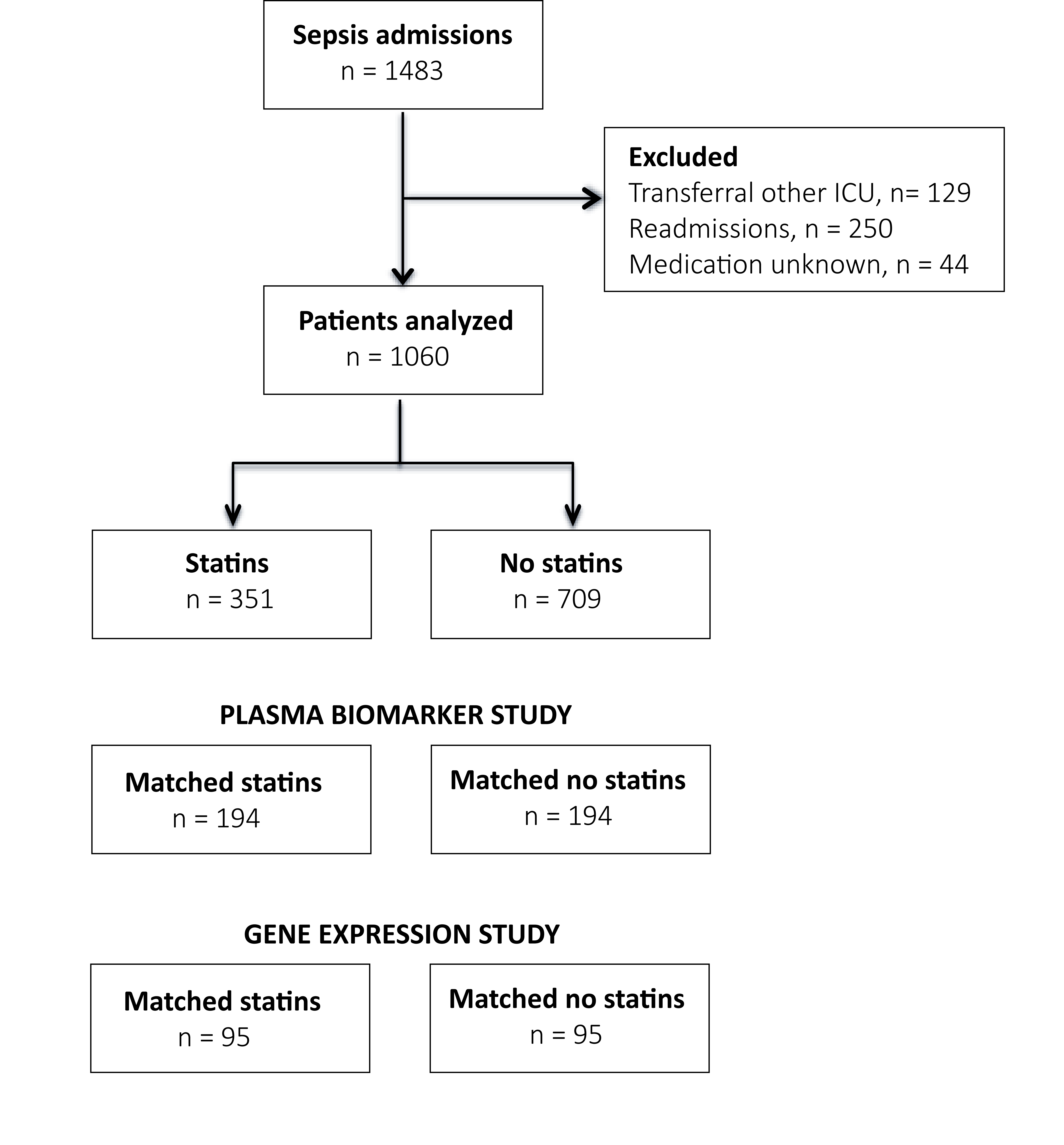


**Supplemental Figure 2. Distribution of propensity scores**

**
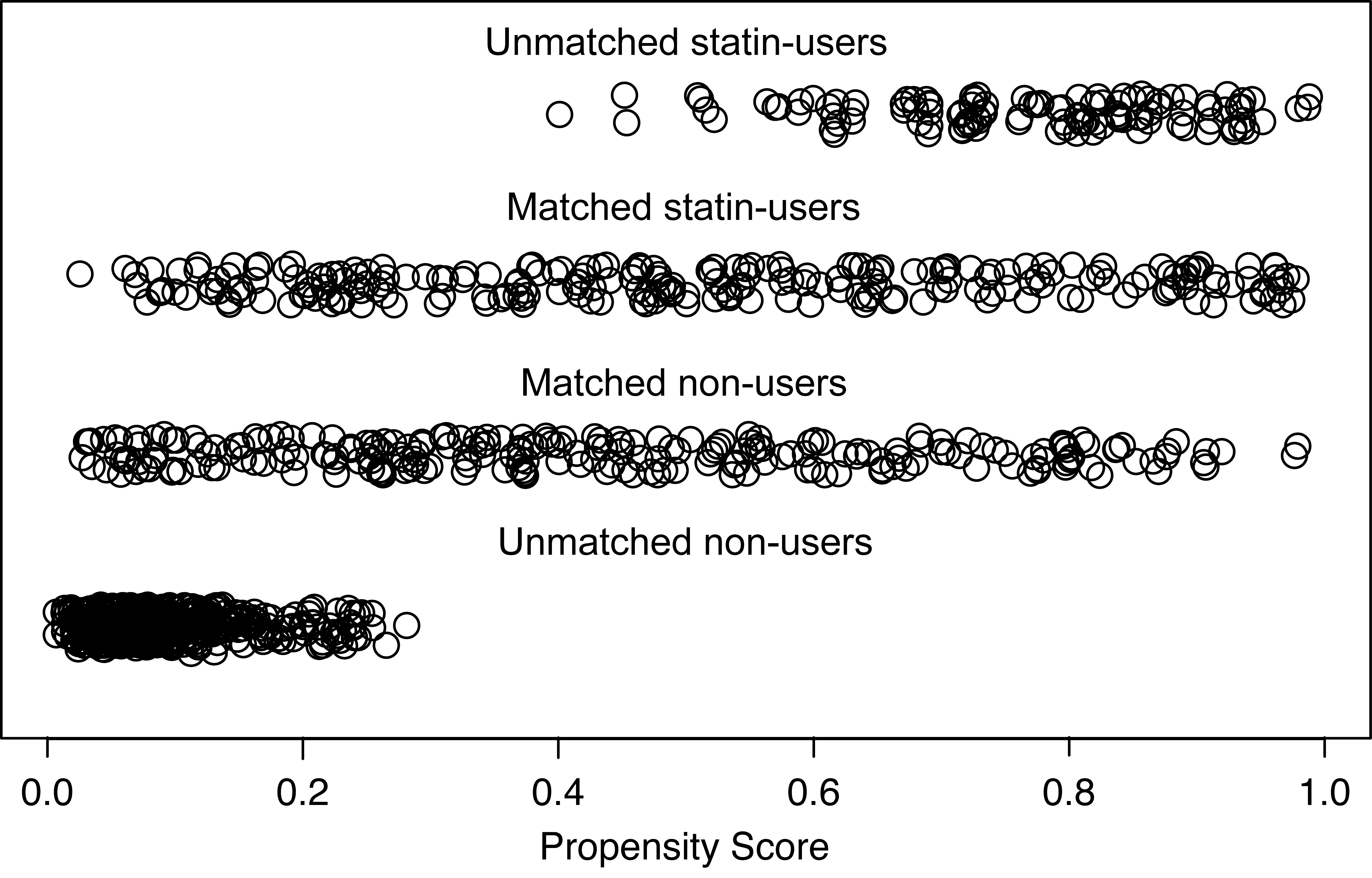
**

**Supplemental Figure 3. Blood transcriptomics of sepsis patients discordant for statin therapy.**

**
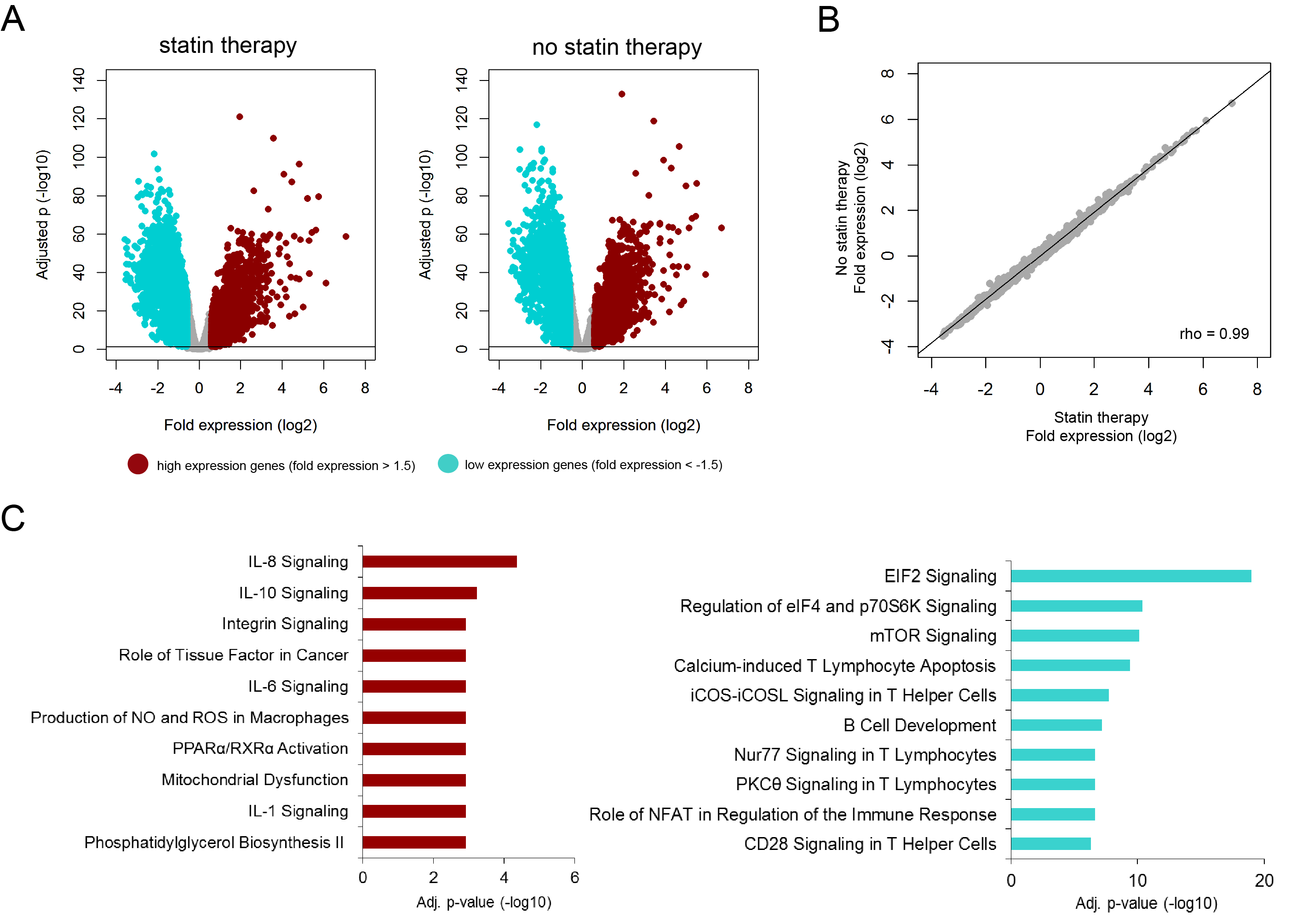
**

(A) Volcano plot representation of differential gene expression in the unmatched cohort of patients treated with statins and patients not treated with statins, both relative to healthy subjects. (B) Dot plot illustrating the strong correlation between expression changes in sepsis patients discordant for statin therapy in the unmatched cohort, relative to healthy subjects. Rho, Spearman’s rho. (C) Ingenuity pathway analysis of genes with elevated expression (red bars) and decreased expression (turquoise).

**Supplemental Table 1.** Host response biomarkers in sepsis patients admitted to

the ICU stratified according to prior use of statins in the unmatched cohort.

|  | Statins  N = 287 *^a^* | No statins  N = 601 | *p* |
| --- | --- | --- | --- |
| **Inflammatory response** *^b^* |  |  |  |
| Interleukin-6 (pg/ml) | 165.2 [47.3-942.2] | 205 [43.6-1265.1] | .46 |
| Interleukin-8 (pg/ml) | 120.6 [51.5-368.7] | 133.2 [49-545.3] | .47 |
| Interleukin-10 (pg/ml) | 11.9 [4.7-46] | 14.3 [4.6-52.4] | .30 |
| MMP-8 (ng/ml) | 3.4 [1.2-10.4] | 3.4 [1.1-11.4] | .69 |
| TIMP-1 (ng/ml) | 584.9 [297.5-1083.7] | 594.9 [268-1213.5] | .64 |
| CRP (mg/l) | 203 [121-301.5] | 212 [115.8-309.8] | .89 |
| **Endothelial cell activation** |  |  |  |
| Soluble ICAM-1 (ng/ml) *^c^* | 172.2 [104.4-294.3] | 199.9 [112-321.3] | .06 |
| Soluble E-Selectin (ng/ml) | 10.8 [5.3-23.3] | 9.5 [4.9-25] | .83 |
| Fractalkine (pg/ml) | 25.5 [13.4-54] | 24 [13.4-60.4] | .94 |
| Angiopoietin-1 (ng/ml) | 2.4 [0.9-5.7] | 2 [0.8-5.7] | .16 |
| Angiopoietin-2 (ng/ml) | 7.5 [3.4-15.3] | 7.3 [3.4-15.3] | .98 |
| Ang-2 / Ang-1 ratio | 2.6 [0.8-10.3] | 3.1 [0.8-13.1] | .51 |
| **Coagulation & Fibrinolysis** |  |  |  |
| Platelets min (x 10^9^/l) | 182 [124-275] | 177 [95-263] | .06 |
| PT max (s) | 16 [13.5-18.5] | 15.7 [13.2-19.1] | .62 |
| aPTT max (s) | 39 [31-54.2] | 39 [31-51] | .48 |
| D-dimer (µg/ml) | 9.2 [4.1-17.6] | 9.4 [4.3-17.5] | .76 |
| Protein C (ng/ml) | 116.4 [91.3-150.9] | 112.7 [84.4-154.1] | .33 |
| Antithrombin (ng/ml) | 707.8 [483.9-989.9] | 754.6 [511.9-1089.7] | .20 |

Results are presented as medians and interquartile ranges.

CRP, C-reactive protein, ICAM-1, intercellular adhesion molecule-1; MMP, matrix metalloproteinase;

TIMP, tissue inhibitor of metalloproteinase.

*^a^* Number of patients of whom plasma was available for measurement of biomarkers.

*^b^* Levels of tumor necrosis factor-α, IL-1β, IL-13, interferon-ɣ and granulocyte-macrophage colony

stimulating factor were undetectable or very low in the vast majority of patients and not different between

groups (data not shown).

*^c^* Soluble intercellular adhesion molecule-1 also originates from leukocytes.

**Table 2. Baseline characteristics of matched statin-users and non-users in the gene expression study**

|  |  | Statins | No statins | *p* |
| --- | --- | --- | --- | --- |
| Characteristics |  | N = 95 | N = 95 |  |
| **Demographics** |  |  |  |  |
| Age, years, mean [SD] |  | 68.6 [9.4] | 68.6 [11.5] | 1 |
| Gender, male (%) |  | 55 (57.9) | 51 (53.7) | .67 |
| Race, white (%) |  | 85 (89.5) | 90 (94.7) | .27 |
| BMI, kg/m2, mean [SD] |  | 26.5 [6.2] | 27.1 [6.7] | .53 |
| **Comorbidities** |  |  |  |  |
| Cerebrovascular disease (%) |  | 16 (16.8) | 14 (14.7) | .84 |
| Chronic cardiovascular insufficiency (%) |  | 9 (9.5) | 6 (6.3) | .61 |
| Chronic renal insufficiency (%) |  | 26 (27.4) | 20 (21.1) | .41 |
| Congestive heart failure (%) |  | 6 (6.3) | 7 (7.4) | 1 |
| COPD (%) |  | 15 (15.8) | 21 (22.1) | .37 |
| Diabetes mellitus (%) |  | 30 (31.6) | 24 (25.3) | .41 |
| Hematologic malignancy (%) |  | 1 (1.1) | - |  |
| Hypertension (%) |  | 38 (40) | 42 (44.2) | .67 |
| Immune deficiency (%) |  | 17 (17.9) | 15 (15.8) | .85 |
| Metastatic malignancy (%) |  | 2 (2.1) | 2 (2.1) | 1 |
| Myocardial infarction (history of) (%) |  | 11 (11.6) | 9 (9.5) | .81 |
| Non-metastatic malignancy (%) |  | 20 (21.1) | 18 (18.9) | .86 |
| Peripheral vascular disease (%) |  | 20 (21.1) | 14 (14.7) | .33 |
| Alcohol or drug abuse (%) |  | 5 (5.3) | 7 (7.4) | .78 |
| **Chronic medication** |  |  |  |  |
| ACE inhibitors and ARBs (%) |  | 39 (41.1) | 42 (44.2) | .77 |
| Anticoagulants (%) |  | 17 (17.9) | 24 (25.3) | .29 |
| Antiplatelet drugs (%) |  | 47 (49.5) | 37 (38.9) | .19 |
| Beta-blockers (%) |  | 57 (60) | 46 (48.4) | .13 |
| Calcium channel blockers (%) |  | 22 (23.2) | 20 (21.1) | .85 |
| Corticosteroids (%) |  | 13 (13.7) | 16 (16.8) | .68 |
| Insulin (%) |  | 17 (17.9) | 13 (13.7) | .56 |
| Oral antidiabetic drugs (%) |  | 21 (22.1) | 16 (16.8) | .46 |
| Other antiarrhythmic drugs (%) |  | 7 (7.4) | 8 (8.4) | 1 |
| Statins |  |  |  |  |
| Simvastatin (%) |  | 50 (52.6) | - |  |
| Atorvastatin (%) |  | 25 (26.3) | - |  |
| Pravastatin (%) |  | 12 (12.6) | - |  |
| Rosuvastatin (%) |  | 5 (5.3) | - |  |
| Fluvastatin (%) |  | 3 (3.2) | - |  |
| **Site of infection** |  |  |  |  |
| Pulmonary (%) |  | 39 (41.1) | 39 (41.1) | 1 |
| Abdominal (%) |  | 19 (20) | 23 (24.2) | .60 |
| Urinary tract (%) |  | 9 (9.5) | 12 (12.6) | .62 |
| Other (%) *^a^* |  | 15 (15.8) | 10 (10.5) | .40 |
| Co-infection (%) |  | 13 (13.7) | 11 (11.6) | .83 |
| Admission type, medical (%) |  | 61 (64.2) | 77 (81.1) | .02 |
| **Causative pathogens** *^b^* |  |  |  |  |
| Gram-positive (%) |  | 48 (50.5) | 34 (35.8) | .06 |
| Gram-negative (%) |  | 58 (61.1) | 71 (74.7) | .23 |
| Yeast/fungi (%) |  | 9 (9.5) | 11 (11.6) | .81 |
| Other (%) |  | 7 (5.1) | 14 (14.7) | ,18 |
| Unknown (%) |  | 15 (10.9) | 13 (13.7) | .71 |
| **Severity of disease in first 24 hours** |  |  |  |  |
| APACHE IV Score, median [IQR] |  | 87 [68-102] | 82 [68-101] | .33 |
| Acute physiology score, median [IQR] |  | 72 [53-86] | 67 [54-83] | .36 |
| SOFA score, median [IQR] *^c^* |  | 8 [5-10] | 7 [5-9] | .07 |
| Organ failure (%) |  | 83 (87.4) | 86 (90.5) | .51 |
| Shock (%) |  | 37 (38.9) | 36 (37.9) | 1 |
| Acute lung injury (%) |  | 26 (27.4) | 31 (32.6) | .53 |
| Acute kidney injury (%) |  | 45 (47.4) | 41 (43.2) | .67 |
| Mechanical ventilation (%) |  | 81 (85.3) | 78 (82.1) | .71 |
| Renal replacement therapy (%) |  | 15 (15.8) | 9 (9.5) | .29 |
| Lactate max. (mmol/l), median [IQR] *^d^* |  | 2.6 [1.7-6.3] | 3.1 [2-7] | .30 |

ACE, angiotensin-converting-enzyme; APACHE, acute physiology and chronic health evaluation; ARBs, angiotensin receptor blockers; BMI, body mass index; COPD, chronic obstructive pulmonary disease; IQR, interquartile range; NSAIDs, non-steroidal anti-inflammatory drugs; SD, standard deviation; SOFA, sequential organ failure assessment.

*^a^* Site of infection: "other" includes cardiovascular infection, mediastinitis and skin infection.

*^b^* Percentages represent the proportion of cases caused by the particular pathogen. In some cases multiple causative pathogens were isolated.

*^c^* Central nervous system not included in score, due to large number of sedated patients

*^d^* Lactate levels were absent in 51 patients

**Table 3. Outcomes of matched statin-users and non-users in the gene expression study**

|  | Propensity-matched cohort | | |
| --- | --- | --- | --- |
|  | Statins | No statins | *p* |
| Outcomes | N = 95 | N = 95 |  |
| Length of stay ICU, median, days [IQR] | 4 [2-10] | 5 [2-10] | .79 |
| Organ failure during admission (%) | 86 (90.5) | 90 (94.7) | 1 |
| Shock during admission (%) | 48 (50.5) | 42 (44.2) | .50 |
| Acute lung injury during admission (%) | 31 (32.6) | 34 (35.8) | .75 |
| Acute kidney injury during admission (%) | 55 (57.9) | 48 (50.5) | .38 |
|  |  |  |  |
| **Mortality** |  |  |  |
| ICU mortality (%) | 22 (23.2) | 23 (24.2) | 1 |
| Hospital mortality (%) | 31 (32.6) | 38 (40) | .36 |
| 30-day mortality (%) | 28 (29.5) | 35 (36.8) | .34 |
| 60-day mortality (%) | 33 (34.7) | 39 (41.1) | .47 |
| 90-day mortality (%) | 41 (43.2) | 41 (43.2) | 1 |

ICU, intensive care unit; IQR, interquartile range
